# Predicting the Potential Distribution of *Haloxylon ammodendron* under Climate Change Scenarios Using Machine Learning of a Maximum Entropy Model

**DOI:** 10.3390/biology13010003

**Published:** 2023-12-20

**Authors:** Fengjin Xiao, Qiufeng Liu, Yun Qin

**Affiliations:** National Climate Center, Chinese Meteorological Administration, Beijing 100081, China; liuqf@cma.gov.cn (Q.L.); qiny@cma.gov.cn (Y.Q.)

**Keywords:** climate change, *H. ammodendron*, Maxent model, potential distribution, prediction

## Abstract

**Simple Summary:**

Global warming has led to changes in species distribution patterns, and climate change directly impacts species’ spatial and temporal distributions, disrupting Earth’s ecological equilibrium. *Haloxylon ammodendron* is a typical desert shrub and plays a vital role in maintaining the structure and function of an arid desert ecosystem. In this study, the Maxent model was employed to predict potentially suitable areas for *H. ammodendron* in the 2030s, 2050s, and 2070s. We found that the suitable distribution area is expected to expand slightly under shared socioeconomic pathway scenarios in the future.

**Abstract:**

*Haloxylon ammodendron (H. ammodendron)* is a second-class protected plant of national significance in China that is known for its growth in desert and semidesert regions, where it serves as a desert ecosystem guardian by playing a substantial role in maintaining ecosystem structure and function. The changing global climate has substantially altered the growth conditions for *H. ammodendron*. This study focuses on identifying the key variables influencing the distribution of *H. ammodendron* and determining their potential impact on future distribution. We employed the Maxent model to evaluate the current climate suitability for *H. ammodendron* distribution and to project its future changes across various shared socioeconomic pathway (SSP) scenarios. Our findings indicate that precipitation during the warmest quarter and precipitation during the wettest month are the most influential variables affecting the potentially suitable habitats of *H. ammodendron*. The highly suitable habitat area for *H. ammodendron* currently covers approximately 489,800 km^2^. The Maxent model forecasts an expansion of highly suitable *H. ammodendron* habitat under all future SSP scenarios, with the extent of unsuitable areas increasing with greater global warming. The increased highly suitable habitats range from 40% (SSP585) to 80% (SSP126) by the 2070s (2060–2080). Furthermore, our results indicate a continued expansion of desertification areas due to global warming, highlighting the significant role of *H. ammodendron* in maintaining desert ecosystem stability. This study offers valuable insights into biodiversity preservation and ecological protection in the context of future climate change scenarios.

## 1. Introduction

Global warming is causing widespread biodiversity loss, ecosystem degradation, and transformations and presents substantial risks to all regions; risks which will intensify with each degree of global warming. In terrestrial ecosystems, 3% to 14% of species face a high risk of extinction at 1.5 °C of global warming, increasing to 3% to 18% at 2 °C [1,2,3]. The nexus between climate and species is central to biology and environmental science. Global warming has led to changes in species distribution patterns [4,5]. Climate change directly impacts species’ spatial and temporal distributions, disrupting Earth’s ecological equilibrium. Climate warming significantly affects the habitats of endangered species, elevating the risk of their extinction [6,7]. Hence, examining the effects of climate change on species distribution is crucial for their preservation and sustainable use.

Over the past century, China’s average temperature has risen by over 2 °C, double the global average increase, with rapid increases in the last 30 years accompanied by severe weather events [8]. Future climate changes will not only alter temperature and precipitation patterns but also impact China’s plant physiology, geographical distribution, and population sizes [9,10,11].

*Haloxylon ammodendron*, a typical desert shrub, possesses physiological and morphological traits that enable it to withstand arid and torrid conditions [12]. This species is indigenous to central Asian desert ecosystems, where it plays a vital role in maintaining arid ecosystem structure and function [13,14]. Worldwide, there are 11 species in the *Haloxylon* genus, with two species in China: *Haloxylon ammodendron* (C. A. Mey.) Bunge and *Haloxylon persicum* Bunge ex Boiss, both belonging to the Chenopodiaceae angiosperm family. It has a flowering period in July and a fruiting period in September. With hard and brittle wood, it thrives in mainland areas exposed to extreme weather conditions [15]. Its leaves become scaly as water content decreases, aiding in drought resistance. *Haloxylon ammodendron* thrives in well-lit, drought-prone conditions, offering a robust root system, with the primary root often exceeding 2 m in depth, which is instrumental in its growth in extreme climates. It exhibits dormancy characteristics, including summer and winter dormancy, which support its growth in harsh climates [16].

*H. ammododendron* predominantly inhabits China’s northwest desert and semidesert regions, mainly on shifting sand dunes and Gobi desert areas [17,18]. Serving as a pioneer species for wind control, sand stabilization, and biological saline-alkali regulation, it contributes to enhanced vegetation, desertification mitigation, reduced wind speed, improved forest microclimates, and the maintenance of desert ecosystem structure and function [19,20]. It also is the host of *Cistanche deserticola*, a precious Chinese herbal medicine known as desert ginseng, which is a second-level endangered species in China. This species is found in Inner Mongolia, Gansu, Ningxia, Qinghai, and Xinjiang at elevations ranging from 150 to 2600 m, with primary distributions in the Junggar Basin, Hashun Gobi, Nuomin Gobi, Alxa High Plain, and Qaidam High Basin. These areas experience an average annual temperature of 2–11 °C, an average January temperature of 18–8 °C, an average July temperature of 22–26 °C, and an annual precipitation of 30–200 mm or even less [21]. Extensive logging and overgrazing have caused severe damage to *Haloxylon ammodendron* forests in China over the past half-century. Consequently, *Haloxylon ammodendron* has been designated as a second-class protected plant at the national level [22,23]. Research on the geographical distribution and resource dynamics of *Haloxylon ammodendron* vegetation in the desert has been a persistent focus [24,25].

The maximum entropy model (Maxent) is a machine learning method widely applied to study species niches by analyzing species–environment relationships using presence-only data and environmental factors [26,27,28,29]. Maxent’s utility arises from its ability to operate with solely the presence of data, enabling diverse applications such as assessing suitable habitats for protected and endangered species [30,31,32], predicting the distribution of invasive species [33], and evaluating distribution shifts driven by climate changes [34]. In this study, the Maxent model was employed to predict potentially suitable areas for *H. ammodendron* in the current period and future time horizons (2030s, 2050s, and 2070s). The study’s objectives include three key aspects: (1) analyzing the impact of climate change on *H. ammodendron*’s distribution range under the future climate change scenarios, (2) identifying the primary environmental variables influencing the potentially suitable distribution of *H. ammodendron*, and (3) forecasting the influence of future climate change on the potential distribution of *H. ammodendron*. In general, our results provide evidence for a better understanding of climate change impact biology distribution and its key climatic factors.

## 2. Materials and Methods

### 2.1. Study Area

**Figure 1:**
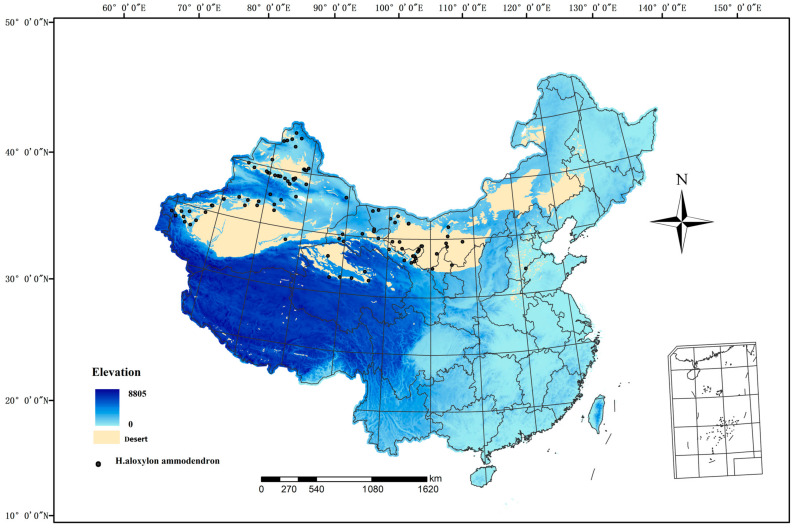
Spatial distribution of occurrence records of *Haloxylon Bunge* in China.

China is afflicted with severe desertification, predominantly concentrated in the northwestern and northern regions. The arid, semiarid, and subhumid arid regions are primarily situated to the west of the Great Khingan Mountains, the northern areas of the Xilingol Plateau and Loess Plateau, the northern regions of the Qilian Mountains, the Qaidam Basin, and the northwestern sector of the Qinghai–Tibet Plateau. This delineates a dry, cold desertification climate zone, including national borders. China exhibits a wide variety of desert types and extensive desertification land, with a combined area of 234.98 × 104 km^2^, constituting 24.48% of the total land area. *H. ammodendron* predominantly thrives in the arid northwestern regions of China, within an inland arid domain spanning 34–48° N and 72–108° E (Figure 1). This region experiences extreme aridity with an annual precipitation of less than 200 mm and a maximum temperature of 47.8 °C, characterizing it as an exceedingly arid and ecologically fragile area. The sparse vegetation type is mainly composed of dry trees, shrubs, and dry succulent plants, *H. ammodendron* is a pioneering species in desert areas of China and plays a vital role in maintaining arid ecosystem structure and function.

### 2.2. Species-Occurrence Records

A total of 120 Chinese *H. ammodendron* occurrence records were obtained from the Global Biodiversity Information Facility (http://www.gbif.org, accessed on 25 March 2023) and an integrated Big BioData infrastructure for CASEarth (https://bio-one.org.cn, accessed on 25 March 2023) (Table S1). We processed the data by selecting records within a 5 km × 5 km range, resulting in 121 valid distribution sites that met the criteria for the Maxent model.

### 2.3. Environment Variables

To predict the species’ future distribution under varying climate change scenarios, we selected 19 environmental variables (Table S2). We utilized CMIP6 datasets for future climate conditions obtained from the World Climate database (https://www.worldclim.org, accessed on 15 April 2023). We chose data with spatial resolutions of 2.5 min to match the scale of the study area. The data included current (1970–2000) and future (2030s, 2050s, 2070s) periods. To simulate *H. ammodendron*’s future distribution, we integrated data from four CMIP6 general circulation models (GCMs): BCC-CSM2-MR, ACCESS-CM^2^, EC-Earth3-Veg, and MIROC6. We also incorporated climate change modeling data for four shared socioeconomic pathways (SSPs): SSP126, SSP245, SSP370, and SSP585. These pathways represent distinct future climate scenarios depending on greenhouse gas emissions. We specifically selected SSP126 (minimal greenhouse gas emissions), SSP245 (moderate greenhouse gas emissions), and SSP585 (high greenhouse gas emissions) to simulate *H. ammodendron*’s suitability distributions in the 2030s (2021–2040), 2050s (2041–2060), and 2070s (2061–2080).

### 2.4. Maxent Model

We utilized the Maxent model version 3.4.4, obtained from http://www.cs.princeton.edu (accessed on 23 December 2022). Maxent estimates the likelihood of species presence by maximizing entropy based on presence records and randomly generated background points. A final distribution model was derived from the average logistic outputs of 10 replicated runs. These outputs yield the probability of presence, ranging from 0 (unlikely) to 1 (highly likely) [26]. Our model incorporated 10 replicate runs with cross-validation, a maximum of 10,000 background points, and a maximum of 1000 iterations. We also activated the ‘remove duplicate presence records’ option, retaining one observation per 30 arc-second grid cell.

The inhomogeneous Poisson process, a commonly employed model, was utilized to describe a random point set *Z* distributed within a given domain *D* [35,36]. When adapting this process for species distribution modeling, we employed the set of occurrence records for *Z*, with *D* representing the geographic study area. This inhomogeneous Poisson process is characterized by an intensity function λ, which assigns a nonnegative real-valued intensity, denoted as *λ*(*z*), to each point *z* within *D*. This intensity function quantifies the likelihood of a point (in this context, an occurrence record of the species) falling at or near *z*. We can establish a probability density over domain *D* as follows:

$$p_{\gamma }\left(z\right)=\gamma (z)/\int_{D}^{\;}\gamma \left(z\right)dz$$

The entropy value of the model is:

$$H=-E_{\gamma }[LN\left(p_{\gamma }\right)]$$

We developed a Maxent model by utilizing the data concerning the current distribution points of *H. ammodendron* and environmental factors as limiting factors. This model involves the ingestion of geographical data pertaining to the *H. ammodendron* population (in.csv format) and environmental variable data, followed by the execution of iterative computations. Subsequently, the *H. ammodendron* population distribution probability within the Chinese regional scale (*D*) is generated as the output.

The training data consisted of 75% randomly selected sample data, while the test data constituted the remaining 25% of the sample data. The habitat suitability curves for each variable were calculated, and the contributions of each variable to the habitat model of *H. ammodendron* were determined using the built-in jackknife test within the software. This jackknife test, which systematically omits each variable, was employed to assess the dominant climatic factors influencing the potential distribution of the species. Additionally, we conducted limiting factor mapping to investigate how the climatic factors that most significantly impact predictions vary spatially across the study area. The receiver operating characteristic (ROC) curve was utilized to describe these findings [37].

### 2.5. Division of the Potentially Suitable Area

Based on the outputs from the Maxent 3.4.4 software, we categorized the suitability of *H. ammodendron* into four grades: high suitability (0.500 ≤ *p* < 1.000), moderate suitability (0.290 ≤ *p* < 0.500), marginal suitability (0.009 ≤ *p* < 0.290), and no suitability (*p* < 0.009) using a natural discontinuous method. According to suitability classification, we employed the ArcGIS 10.5 grid calculation tool to compute the suitable area. We defined the mixed area as the medium-to-highly suitable growing regions (26~100%) of *H. ammodendron*, drew the common distribution area, and used the ArcGIS raster calculation tool to compute this mixed area. Likewise, we utilized the “Zonal Geometry” tool in ArcGIS 10.5 to reduce the suitable area of *H. ammodendron* to a central point under both current and future climate change scenarios. The shift in the position of this point reflects the geographical migration process of the two species within the suitable area. Subsequently, we calculated the centroid migration distances of *H. ammodendron* across different time periods and climate scenarios.

## 3. Results

### 3.1. Model Performance and AUC

In each iteration of the training algorithm, we augmented the contribution of the corresponding variable with the increase in regularized gain or subtracted it from it if the change in the absolute value of lambda was negative. For the second estimate, we randomly permuted each environmental variable’s values on training presence and background data. We adopted the receiver operating characteristic (ROC) curve to assess the performance of the maximum entropy model. The area under the ROC curve (AUC) is an independent threshold measure of model performance, indicating the model’s ability to differentiate between presence and background data. AUC values range from 0 to 1, with higher values signifying more accurate predictions.

We also re-evaluated the model on the permuted data, displaying the resultant decrease in training AUC in Figure S1. Variable contributions should be interpreted with caution in cases where predictor variables are correlated. The results indicate that the accuracy of the Maxent model simulations during both the current period and future climate change scenarios is “excellent”. The average AUC is 0.947, with a maximum AUC value of 0.954 in SSP585–2070s and a minimum AUC value of 0.939 in SSP126–2070s. These results affirm the high reliability of the simulations when analyzing the impact of climate change on the distribution of *H. ammodendron* in China.

### 3.2. Analysis of Variable Contributions

**Figure 2:**
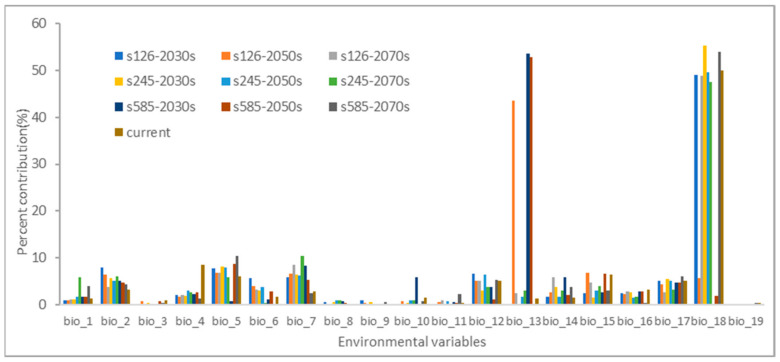
The percentage contributions for environmental variables in the Maxent model.

In Figure 2, estimations of the relative contributions of environmental factors to the Maxent model can be found. These factors have a significant impact on the distribution of *H. ammodendron*. Out of the 19 environmental factors considered, the following factors made noteworthy contributions: precipitation of the warmest quarter (bio_18), with an average contribution of 36.19%; precipitation of the wettest month (bio_13), with an average contribution of 15.88%; max temperature of the warmest month (bio_5), with an average contribution of 6.93%; temperature annual range (bio_7), with an average contribution of 6.33%; mean diurnal range (bio_2), with an average contribution of 5.24%; precipitation of the driest quarter (bio_17), with an average contribution of 4.69%; annual precipitation (bio_12), with an average contribution of 4.58%; and precipitation seasonality (bio_15), with an average contribution of 4.25%. Together, these eight environmental factors contribute to a total of 83.99%, signifying their significance in the distribution of *H. ammodendron*.

The jackknife test results also indicate that the key variables include precipitation of the warmest quarter (bio_18), precipitation of the wettest month (bio_13), max temperature of the warmest month (bio_5), temperature annual range (bio_7), and mean diurnal range, as illustrated in Figure S2.

**Figure 3:**
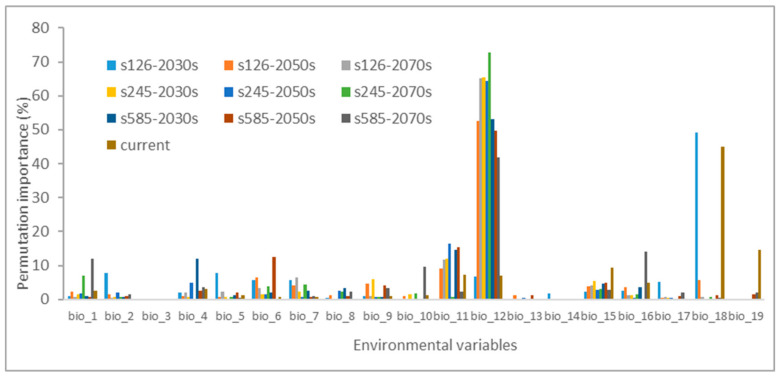
The permutation importance for environmental variables in the Maxent model.

Additionally, it is noteworthy that annual precipitation (bio_12) and mean temperature of the coldest quarter (bio_11), along with precipitation of the warmest quarter (bio_18), precipitation seasonality (bio_15), and minimum temperature of the coldest month (bio_6), displayed notably higher importance during the permutation analysis (Figure 3). This underscores the critical roles of precipitation and temperature when predicting the likely distribution of *H. ammodendron*.

### 3.3. Potentially Suitable Area under Current and Future Scenarios

**Figure 4:**
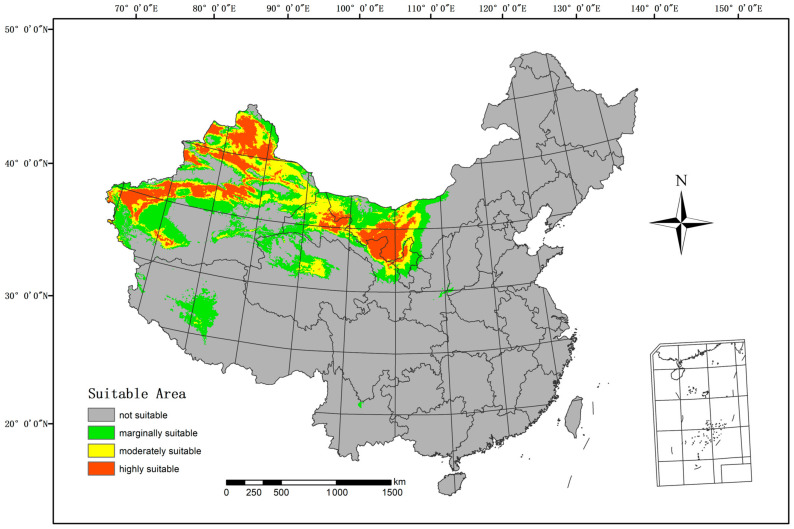
Current suitable climatic distribution of *H. ammodendron* in China.

The Maxent model was employed to forecast the suitable regions for *H. ammodendron* in the current climate, as depicted in Figure 4. The highly suitable area spans 489,800 km^2^, constituting 5.10% of China’s total land area. The moderately suitable area includes 562,600 km^2^, equivalent to 5.86% of China’s land area. The marginally suitable area covers 780,700 km^2^, accounting for 8.13% of China’s land area. These regions include Xinjiang, Gansu, Ningxia, Inner Mongolia, Tibet, Qinghai, and other parts of China.

**Figure 5:**
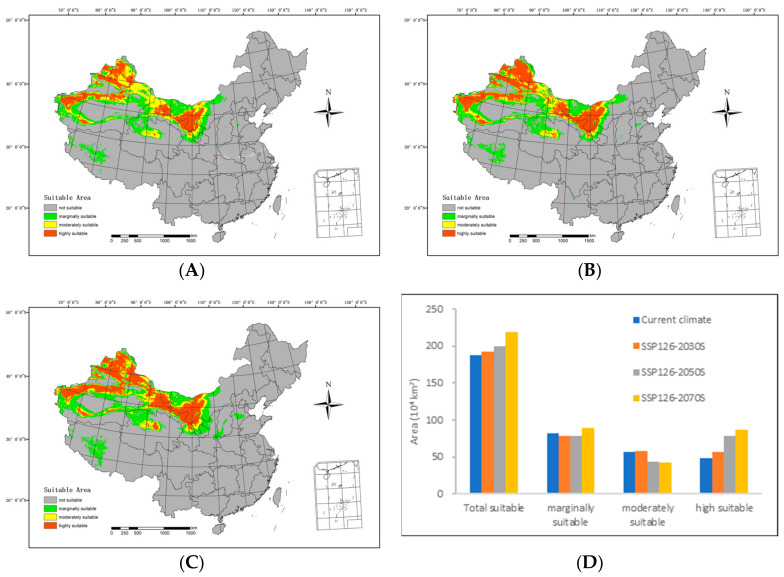
Potential suitable distributions and areas of *H. ammodendron* in China under the SSP126 shared socioeconomic pathway scenarios, including (**A**) SSP126-2030s, (**B**) SSP126-2050s, (**C**) SSP126-2070s, and (**D**) area changes.

In the future high-emission scenario (SSP126), the extent of suitable areas within the country generally exhibits an upward trajectory when compared with the prevailing climatic conditions (Figure 5).

By 2030S, the area classified as highly suitable will include 564,200 km^2^, while the moderately suitable area will span 578,700 km^2^. The marginally suitable area will extend over 781,700 km^2^, resulting in a total suitable area of 192.46 million km^2^. In comparison with the range of suitable areas under current climatic conditions, there is an increase of 74,400 km^2^ in the highly suitable areas and 16,100 km^2^ in the moderately suitable areas. However, the marginally suitable areas have diminished by 35,000 km^2^, resulting in an overall increment of 55,500 km^2^.

By 2050S, the area of highly suitable area will cover 782,600 km^2^, with the moderately suitable area spanning 431,400 km^2^. The marginally suitable area will include 784,100 km^2^, culminating in a total suitable area of 1.981 million km^2^. In contrast with the range of suitable areas under current climatic conditions, the moderately suitable areas have reduced by 131,200 km^2^, and the marginally suitable areas have shrunk by 32,600 km^2^. Simultaneously, the highly suitable areas have expanded by 292,800 km^2^, resulting in an overall increase of 12,900 km^2^.

Regarding the 2070s, the highly suitable area is anticipated to span 873,000 km^2^, with the moderately suitable area covering 429,000 km^2^. The marginally suitable area is predicted to extend over 889,400 km^2^, resulting in a total suitable area of 2,191,300 km^2^. Compared with the range of suitable areas under the current climatic conditions, there is a considerable augmentation of 383,200 km^2^ in the highly suitable areas and 72,700 km^2^ in the marginally suitable areas. Conversely, the moderately suitable areas have contracted by 133,600 km^2^, leading to a net increase of 322,200 km^2^.

**Figure 6:**
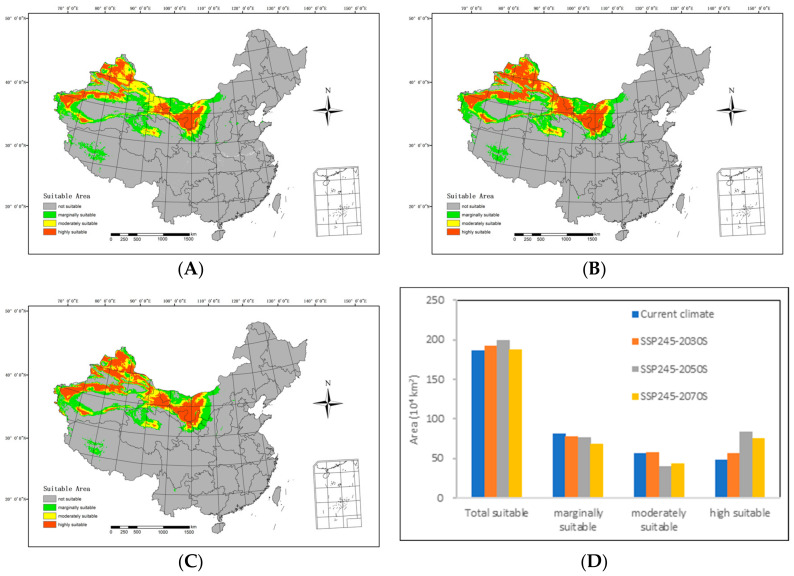
An overview of the potential suitable distributions and areas of *H. ammodendron* in China in the future under the SSP245 shared socioeconomic pathway scenarios, including (**A**) SSP245-2030s, (**B**) SSP245-2050s, (**C**) SSP245-2070s, and (**D**) area changes.

In the future medium-emission scenario (SSP245), the extent of suitable areas within the country generally follows an ascending trend compared with the current climatic conditions, as depicted in Figure 6.

By the 2030s, the highly suitable area is anticipated to include 554,000 km^2^, with the moderately suitable area extending over 578,900 km^2^. The marginally suitable area is projected to cover 787,700 km^2^, resulting in a total suitable area of 1,913,600 km^2^. In relation to the range of suitable areas under the current climatic conditions, there is an expansion of 64,200 km^2^ in the highly suitable areas and 16,300 km^2^ in the moderately suitable areas. However, the marginally suitable areas have contracted by 36,600 km^2^, resulting in a net increase of 44,500 km^2^.

Moving forward to the 2050s, the highly suitable area is expected to span 833,300 km^2^, while the moderately suitable area will include 398,700 km^2^. The marginally suitable area is predicted to cover 76,400 km^2^, contributing to a total suitable area of 1.995 million km^2^. In comparison with the range of suitable areas under the current climatic conditions, there is a decrease of 164,600 km^2^ in the moderately suitable areas and 52,700 km^2^ in the less suitable areas. Simultaneously, the highly suitable areas expanded by 343,500 km^2^, resulting in an overall increase of 126,100 km^2^.

Looking ahead to the 2070s, the highly suitable area is predicted to extend over 770,700 km^2^, while the moderately suitable area will span 4410 km^2^. The marginally suitable area is estimated to cover 681,300 km^2^, resulting in a total suitable area of 1.88 million km^2^. In comparison with the range of suitable areas under current climatic conditions, there is a reduction of 121,700 km^2^ in the moderately suitable areas and 135,400 km^2^ in the less suitable areas. Simultaneously, the highly suitable areas expanded by 270,900 km^2^, leading to a net increase of 12,900 km^2^.

**Figure 7:**
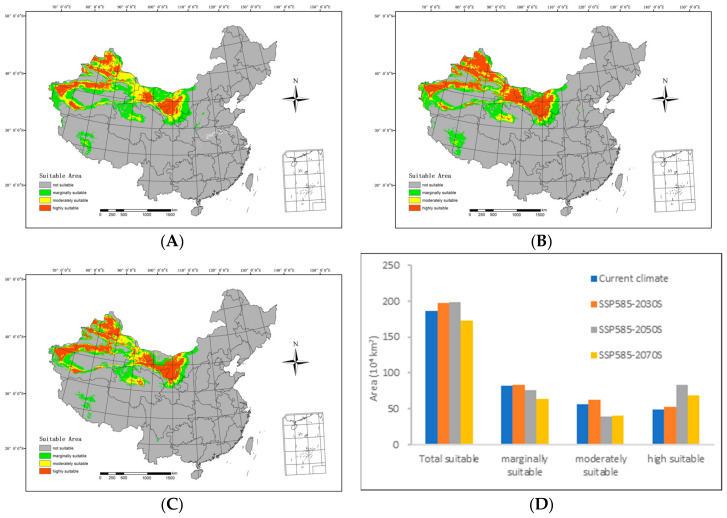
Potential suitable distributions and areas of *H. ammodendron* in China in the future under SSP585 shared socioeconomic pathway scenarios, including (**A**) SSP585–2030s, (**B**) SSP585–2050s, (**C**) SSP585–2070s, and (**D**) area changes.

In the future low-emission scenario (SSP585), the extent of suitable areas within the country is expected to initially increase and then decrease compared with the current climatic conditions, as illustrated in Figure 7.

By the 2030s, the highly suitable area is projected to span 523,100 km^2^, with the moderately suitable area covering 617,700 km^2^. The marginally suitable area is anticipated to extend over 837,500 km^2^, resulting in a total suitable area of 1,978,300 km^2^. In relation to the range of suitable areas under current climatic conditions, there is an expansion of 50,500 km^2^ in the highly suitable areas and 20,800 km^2^ in the medium suitable areas. The overall increase amounts to 109,200 km^2^.

Moving forward to the 2050s, the highly suitable area is expected to cover 830,700 km^2^, while the moderately suitable area will include 395,800 km^2^. The marginally suitable area is projected to span 755,600 km^2^, contributing to a total suitable area of 1,982,100 km^2^. In comparison with the range of suitable areas under the current climatic conditions, there is a reduction of 61,000 km^2^ in the low suitable areas and 166,900 km^2^ in the medium suitable areas. Simultaneously, the highly suitable areas expanded by 340,900 km^2^, resulting in an overall increase of 113,000 km^2^.

Looking ahead to the 2070s, the highly suitable area is predicted to extend over 687,700 km^2^, with the moderately suitable area spanning 407,100 km^2^. The marginally suitable area is estimated to cover 637,700 km^2^, leading to a total suitable area of 1,731,800 km^2^. In relation to the range of suitable areas under the current climatic conditions, there is a decrease of 179,700 km^2^ in the low suitable areas and 155,500 km^2^ in the medium suitable areas. Simultaneously, the highly suitable areas expanded by 197,900 km^2^, resulting in a net decrease of 137,300 km^2^.

## 4. Discussion

The reliability of simulation results for the species distribution model largely depends on the sampling range and sample size of the population. The larger the population sample size collected, the wider the region covered, the richer the information about the relationship between species and the environment obtained, the more constraints the Maxent model has to establish, and the higher the accuracy of species distribution model estimation [38,39]. The AUC value for the area under the ROC curve is recognized as the best measure of model prediction accuracy [40]. In this study, the average AUC values of environmental variables for the current climate condition and nine future SSPs were 0.921 for the verification set, indicating that the sampling range of population distribution points and the scale of the study area will impact the simulation results of the Maxent model. This study is based on population samples from the entire distribution area of *H. ammodendron,* which can represent the habitat of its distribution area and fundamentally avoid deviations in simulation results due to sample issues.

The total suitable distribution area of *H. ammodendron* in the research area will show an increasing trend in the future, mainly due to a significant increase in highly suitable distribution areas. Unlike the highly suitable distribution areas in the Tarim Basin, the Qaidam Basin has seen a slight decrease. In arid areas with harsh environmental conditions, *H. ammodendron*, as a flagship plant in the desert, has adapted to environmental conditions during its long-term evolution [41]. *Haloxylon ammodendron* is mainly distributed in the desert areas of northwest China, which is located in the Eurasian continent’s hinterland and is one of the world’s driest regions at the same latitude. It is sensitive to global climate change and is a fragile ecological environment. It is also the primary region of desert distribution in China. In the 1980s, the climate in the northwest region of China (desertification area) shifted from warm and dry to warm and humid [42,43], which is conducive to the growth of desert plants.

However, previous research indicates that, in the last 50 years, the temperature in northwestern China has exhibited a rising trend, with the warming season mainly occurring in the winter half-year. Due to global warming, the spring climate in the northwestern desert has significantly shortened, and the dry and hot summer climate in the desert arrives earlier. Although this process is variable, the shallow desert soil dominated by sandy soil is rapidly drying out, leading to the rapid death of *H. ammodendron*, a common phenomenon in desert vegetation [44]. Climate change has resulted in sharp temperature variations and reduced rainfall in arid areas in the early spring, leading to the death of a large number of *H. ammodendron* seedlings [45,46].

Arid and semiarid areas in China span 53% of the land area. Measures to curb desertification and alleviate its impacts on crops, pastures, and human life have been developed and successfully implemented in China [47,48]. Cultivating sand-stabilizing plants is among the most important and widely used sandbreak systems, with cultivated sandbreaks covering a total of 47,600 km^2^. *H. ammodendron* has the ecological functions of windbreak and sand fixation, soil improvement, microclimate improvement, and biodiversity maintenance, playing a significant role in maintaining and building the ecological environment. Overgrazing, prolonged deforestation, woodcutting, rat infestation, powdery mildew, and extreme drought have reduced the variety and amount of vegetation, resulting in the degradation and death of the forest. *H. ammodendron* serves as the host for the rare medicinal plant Cistanche. Due to unrestrained development and utilization, the wild resources of Cistanche are depleting, and it has been listed as a national second-class protected plant by the state. It is also included in the International List of Wild Plant Protection [49]. The established national Nature Reserve of *H. ammodendron* Forest in Qinghai and the White *H. ammodendron* Forest Nature Reserve in Ganjiahu, Xinjiang play crucial roles in protecting, developing, and rationalizing *H. ammodendron* species.

The selection of environmental variables is key to assessing habitat suitability for a species. Factors such as climate, soil, topography, hydrology, biology, and human activities comprehensively restrict species’ distribution range [50]. The reliability of habitat suitability assessment results depends on the representativeness and completeness of the selected environmental variables [51]. This study’s advantage is that it provides scientific information for predicting *H. ammodendron* distribution in the future. However, there are some limitations. First, our model prediction is based on data restrictions, and we did not include other variables that may affect the distribution of *H. ammodendron*.

The simulation of species distribution is based on three assumptions. First, that species’ ecological niches are conservative; second, the migration ability of a species is infinite; and third, that species’ ecological needs and distributions are balanced. Under these three assumptions, it is more reasonable to project the model into future time periods to predict changes in species’ distribution range. The model’s establishment does not consider biological factors such as species’ migration ability, geographical barriers, and interspecies competition [52].

## 5. Conclusions

This study employed the Maxent model to investigate and predict the potentially suitable habitats of *H. ammodendron* under different climate change scenarios. The model effectively simulated the distribution range of *H. ammodendron* in China. Environmental factors, such as precipitation of warmest quarter (Bio_18), precipitation of wettest month (Bio_13), max temperature of warmest month (Bio_5), temperature annual range (Bio_7), and mean diurnal range (mean of monthly (max temp–min temp)) (Bio_2), significantly impacted *H. ammodendron* survival and distribution. The predicted areas that are potentially suitable for *H. ammodendron* under historical climate conditions include 1.87 million km^2^. Over time, the core distribution area of *H. ammodendron* remains concentrated in regions such as Xinjiang, Gansu, Qinghai, Ningxia, Inner Mongolia, and Tibet. In the 2030s, 2050s, and 2070s, the suitable distribution area, including marginally suitable, moderately suitable, and highly suitable areas, is expected to expand slightly. Notably, the highly suitable area is set to expand significantly, while the marginally and moderately suitable areas may contract under various future climate change scenarios. It is imperative to implement effective conservation measures to protect *H. ammodendron*’s core distribution areas in response to climate warming and anthropogenic activities.

## Data Availability

The data presented in this study are available from the authors upon request.

## Supplementary Materials

The following supporting information can be downloaded at www.mdpi.com/xxx/s1. Table S1: The setting points in this experiment; Table S2: Definitions of the 19 bioclimatic variables; Figure S1: ROC curve values and AUC under current climate conditions and various future shared socioeconomic pathway scenarios; Figure S2: The jackknife test, assessing the training gains associated with environmental variables under various conditions.
