# Supplementary material for: Predicting the Potential Distribution of Haloxylon ammodendron under Climate Change Scenarios Using Machine Learning of a Maximum Entropy Model
Source: Biology (Basel). 2023 Dec 20;13(1):0. doi: 10.3390/biology13010003 (PMC11154351; doi:10.3390/biology13010003)
Supplement: Supplementary file 1 [file biology-13-00003-s001.zip › Figure S1 ROC curve values and AUC under current climate conditions and various future shared socioeconomic pathway scenario.pdf]

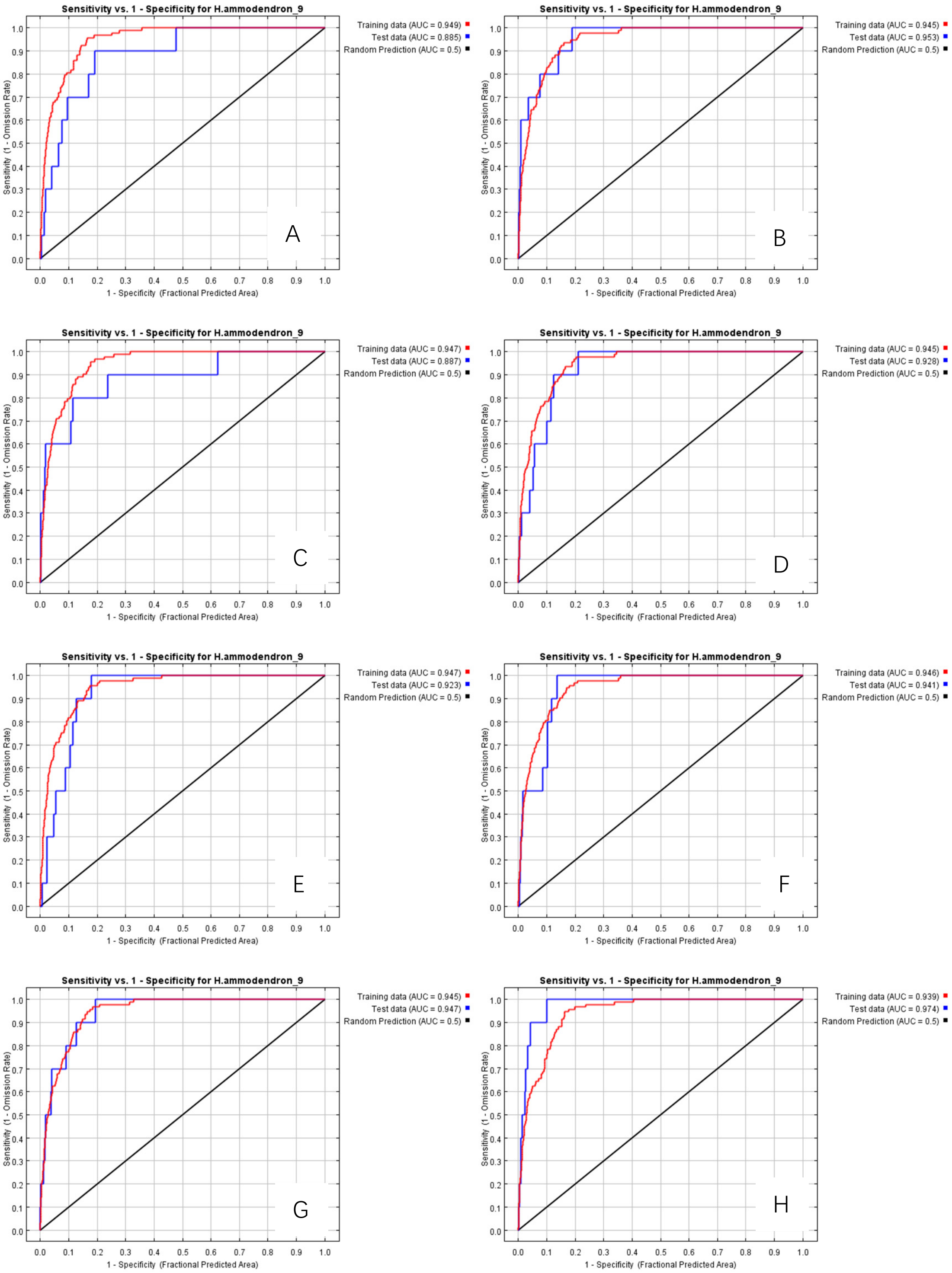
Sensitivity vs. 1 - Specificity for H.ammodendron_9
Training data (AUC = 0.949)
Test data (AUC = 0.885)
Random Prediction (AUC = 0.5)
Sensitivity (1 - Omission Rate)
1 - Specificity (Fractional Predicted Area)
A
Sensitivity vs. 1 - Specificity for H.ammodendron_9
Training data (AUC = 0.945)
Test data (AUC = 0.953)
Random Prediction (AUC = 0.5)
Sensitivity (1 - Omission Rate)
1 - Specificity (Fractional Predicted Area)
B
Sensitivity vs. 1 - Specificity for H.ammodendron_9
Training data (AUC = 0.947)
Test data (AUC = 0.887)
Random Prediction (AUC = 0.5)
Sensitivity (1 - Omission Rate)
1 - Specificity (Fractional Predicted Area)
C
Sensitivity vs. 1 - Specificity for H.ammodendron_9
Training data (AUC = 0.945)
Test data (AUC = 0.928)
Random Prediction (AUC = 0.5)
Sensitivity (1 - Omission Rate)
1 - Specificity (Fractional Predicted Area)
D
Sensitivity vs. 1 - Specificity for H.ammodendron_9
Training data (AUC = 0.947)
Test data (AUC = 0.923)
Random Prediction (AUC = 0.5)
Sensitivity (1 - Omission Rate)
1 - Specificity (Fractional Predicted Area)
E
Sensitivity vs. 1 - Specificity for H.ammodendron_9
Training data (AUC = 0.946)
Test data (AUC = 0.941)
Random Prediction (AUC = 0.5)
Sensitivity (1 - Omission Rate)
1 - Specificity (Fractional Predicted Area)
F
Sensitivity vs. 1 - Specificity for H.ammodendron_9
Training data (AUC = 0.945)
Test data (AUC = 0.947)
Random Prediction (AUC = 0.5)
Sensitivity (1 - Omission Rate)
1 - Specificity (Fractional Predicted Area)
G
Sensitivity vs. 1 - Specificity for H.ammodendron_9
Training data (AUC = 0.939)
Test data (AUC = 0.974)
Random Prediction (AUC = 0.5)
Sensitivity (1 - Omission Rate)
1 - Specificity (Fractional Predicted Area)
H

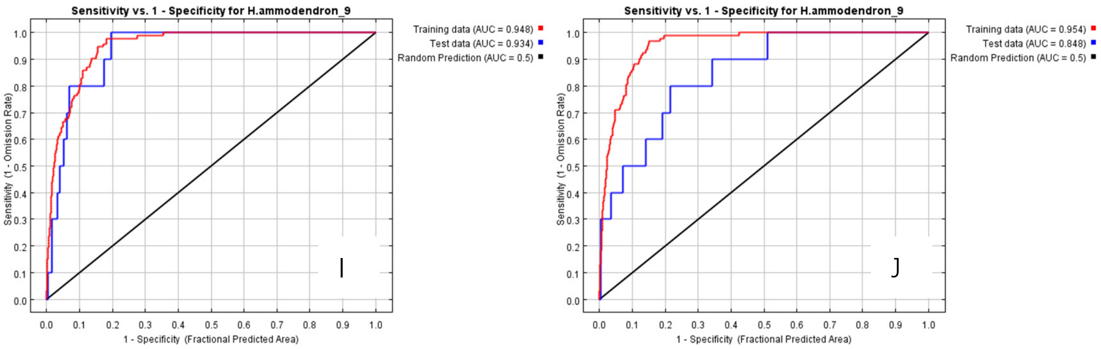

Figure S1. ROC curve values and AUC under current climate conditions and various future shared socioeconomic pathway scenarios: (A) Current climate conditions, (B) SSP126–2030s, (C) SSP126–2050s, (D) SSP126–2070s, (E) SSP245–2030s, (F) SSP246–2050s, (G) SSP245–2070s, (H) SSP585–2030s, (I) SSP585–2050s, (J) SSP585–2070s.
