# Supplementary material for: Predicting the Potential Distribution of Haloxylon ammodendron under Climate Change Scenarios Using Machine Learning of a Maximum Entropy Model
Source: Biology (Basel). 2023 Dec 20;13(1):0. doi: 10.3390/biology13010003 (PMC11154351; doi:10.3390/biology13010003)
Supplement: Supplementary file 1 [file biology-13-00003-s001.zip › Figure S2. The jackknife test, assessing the training gains associated with environmental variables under various conditions.pdf]

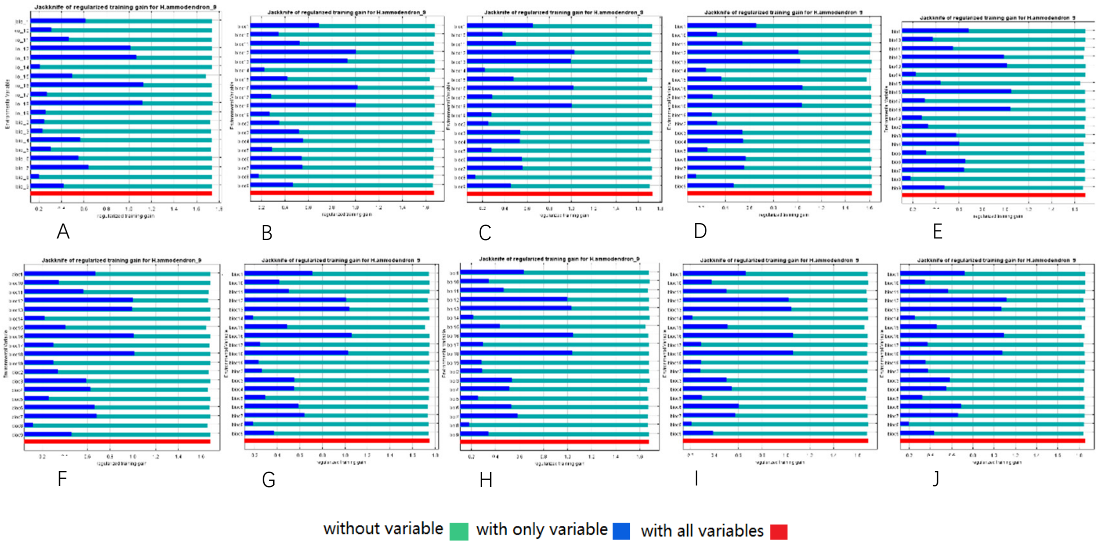

Figure S2. The jackknife test, assessing the training gains associated with environmental variables under various conditions: (A) Current climate conditions, (B) SSP126–2030s, (C) SSP126–2050s, (D) SSP126–2070s, (E) SSP245–2030s, (F) SSP246–2050s, (G) SSP245–2070s, (H) SSP585–2030s, (I) SSP585–2050s, and (J) SSP585–2070s.
