# Supplementary figures and images for: Predicting the Potential Distribution of Haloxylon ammodendron under Climate Change Scenarios Using Machine Learning of a Maximum Entropy Model
Source: Biology (Basel). 2023 Dec 20;13(1):0. doi: 10.3390/biology13010003 (PMC11154351; doi:10.3390/biology13010003)

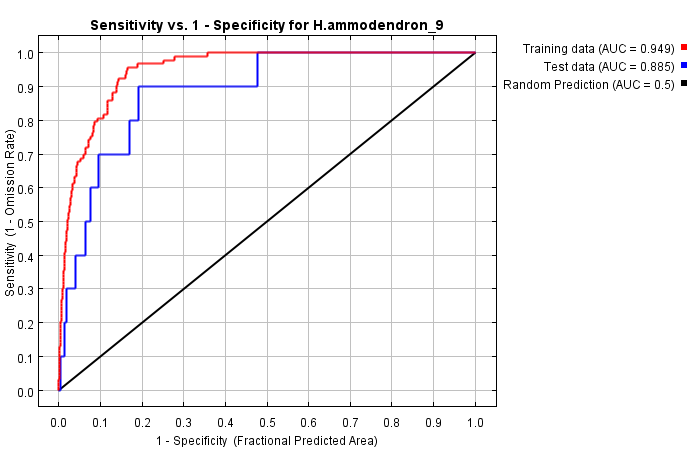

Supplement: Supplementary file 1 [file biology-13-00003-s001.zip › Figure S1 AUC/current_roc.png]

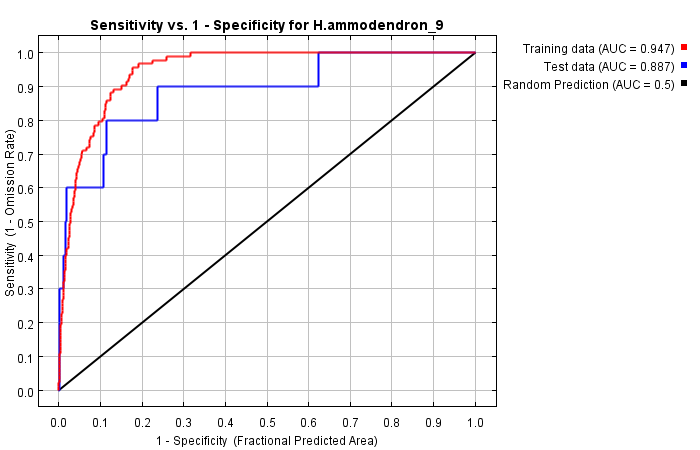

Supplement: Supplementary file 1 [file biology-13-00003-s001.zip › Figure S1 AUC/ssp126_30_roc.png]

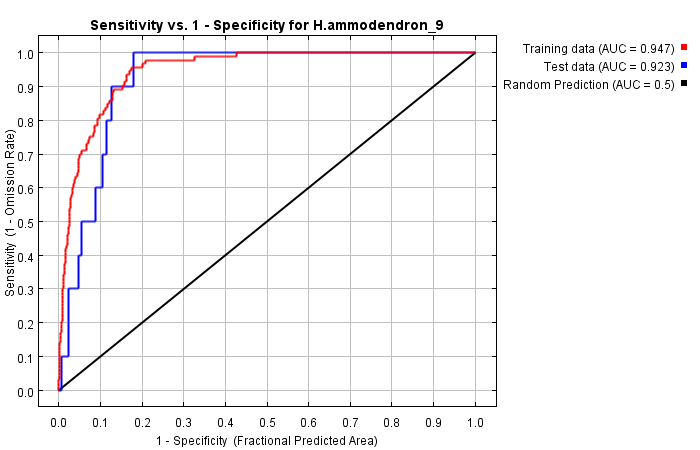

Supplement: Supplementary file 1 [file biology-13-00003-s001.zip › Figure S1 AUC/ssp126_50_roc.png]

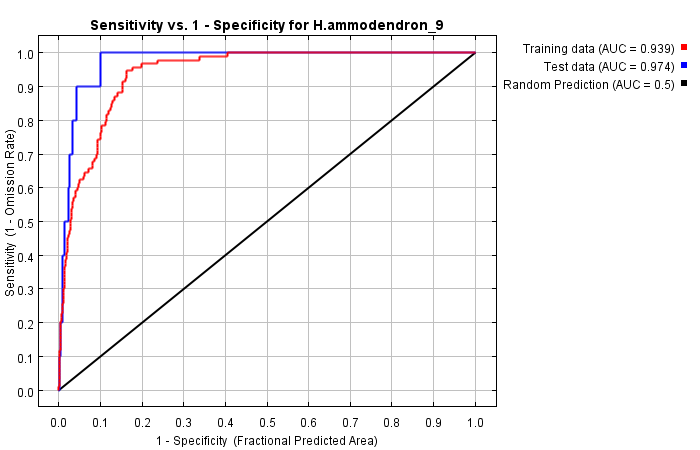

Supplement: Supplementary file 1 [file biology-13-00003-s001.zip › Figure S1 AUC/ssp126_70_roc.png]

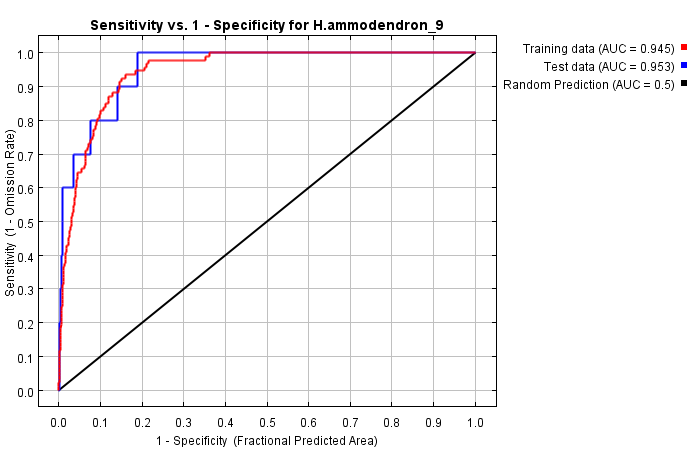

Supplement: Supplementary file 1 [file biology-13-00003-s001.zip › Figure S1 AUC/SSP245_30_roc.png]

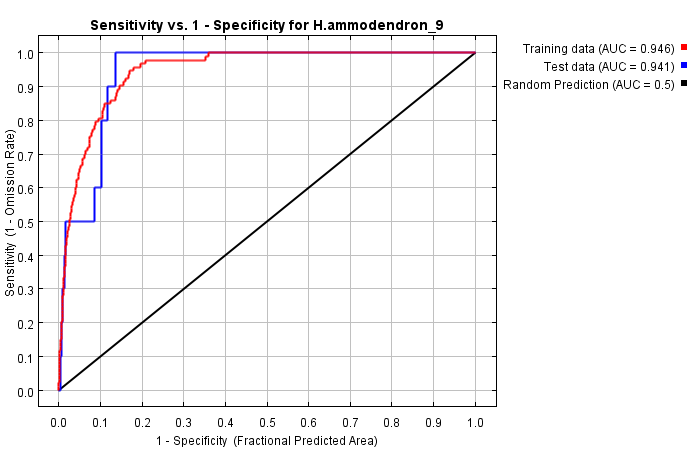

Supplement: Supplementary file 1 [file biology-13-00003-s001.zip › Figure S1 AUC/ssp245_50_roc.png]

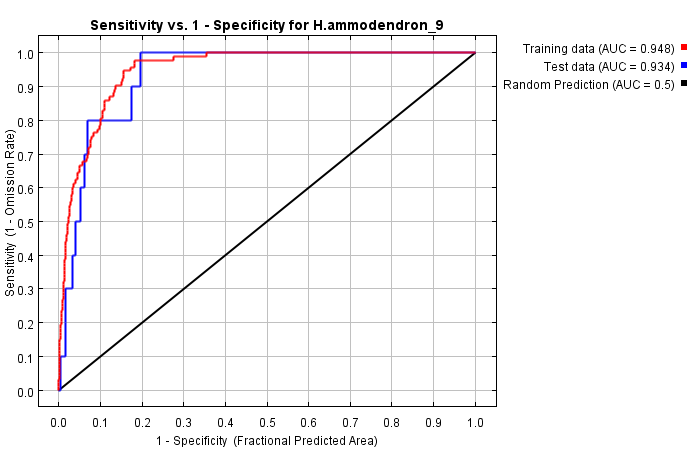

Supplement: Supplementary file 1 [file biology-13-00003-s001.zip › Figure S1 AUC/ssp254_70_roc.png]

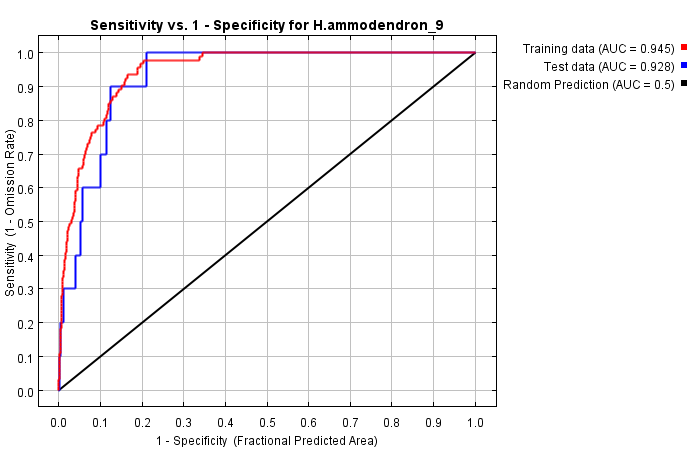

Supplement: Supplementary file 1 [file biology-13-00003-s001.zip › Figure S1 AUC/ssp585_30_roc.png]

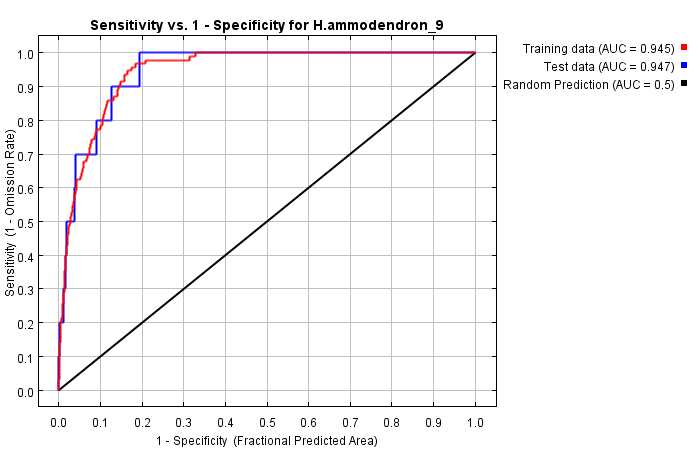

Supplement: Supplementary file 1 [file biology-13-00003-s001.zip › Figure S1 AUC/ssp585_50_roc.png]

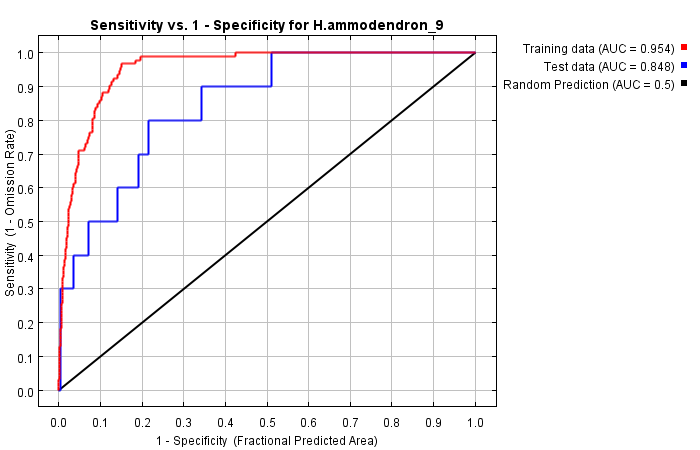

Supplement: Supplementary file 1 [file biology-13-00003-s001.zip › Figure S1 AUC/ssp585_70_roc.png]

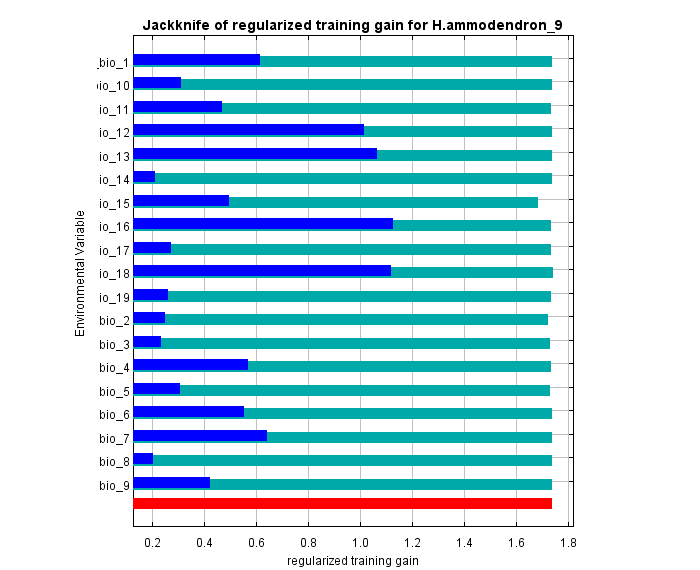

Supplement: Supplementary file 1 [file biology-13-00003-s001.zip › Figure S2 Jackknife/current_jacknife.png]

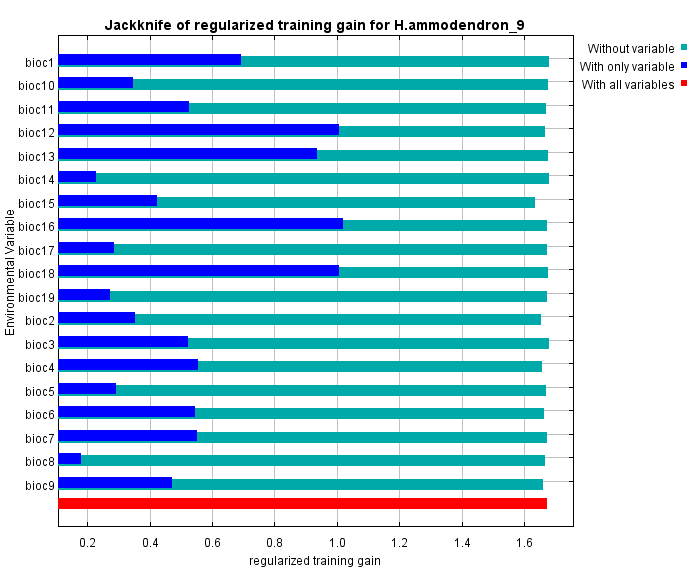

Supplement: Supplementary file 1 [file biology-13-00003-s001.zip › Figure S2 Jackknife/S126_-30_jacknife.png]

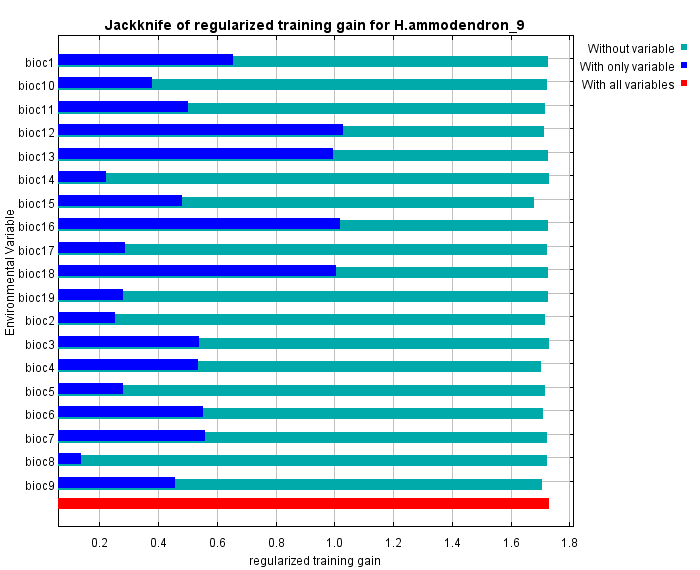

Supplement: Supplementary file 1 [file biology-13-00003-s001.zip › Figure S2 Jackknife/S126_50_jacknife.png]

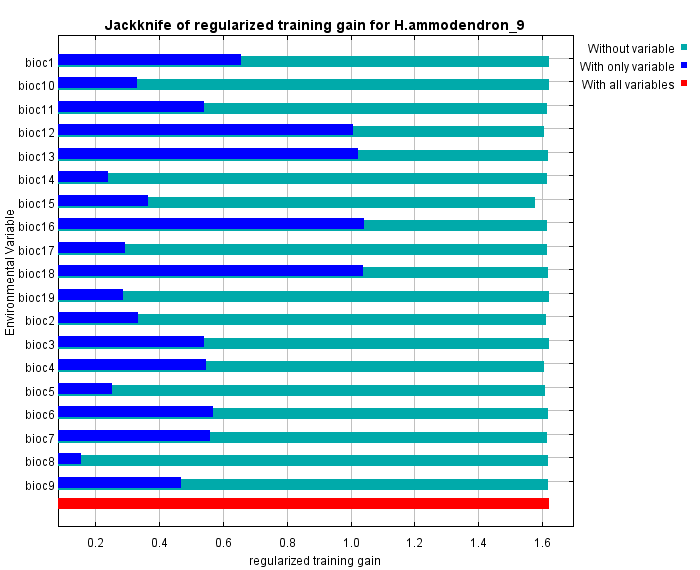

Supplement: Supplementary file 1 [file biology-13-00003-s001.zip › Figure S2 Jackknife/S126_70_jacknife.png]

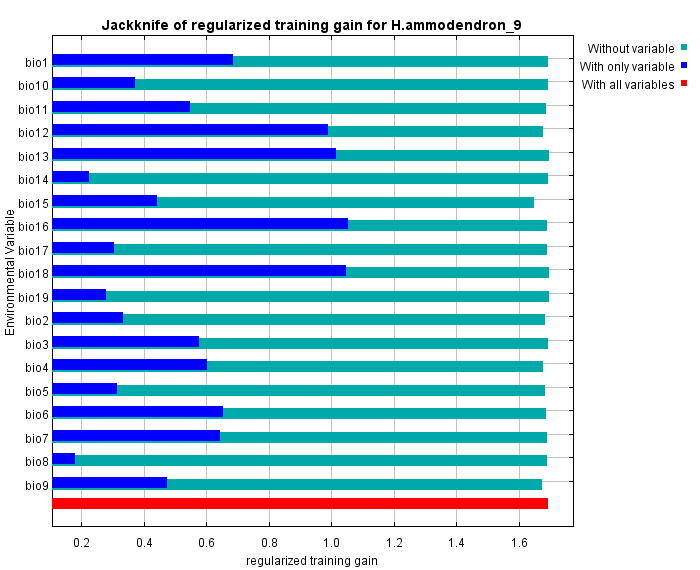

Supplement: Supplementary file 1 [file biology-13-00003-s001.zip › Figure S2 Jackknife/s245_30_jacknife.png]

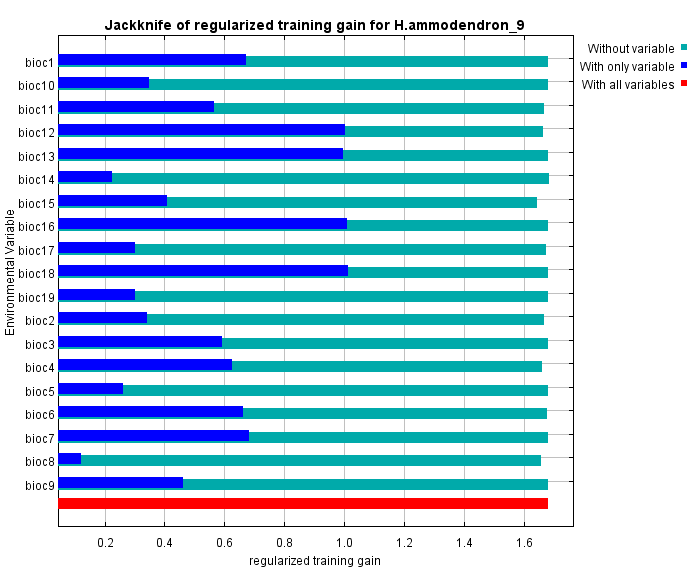

Supplement: Supplementary file 1 [file biology-13-00003-s001.zip › Figure S2 Jackknife/S245_50_jacknife.png]

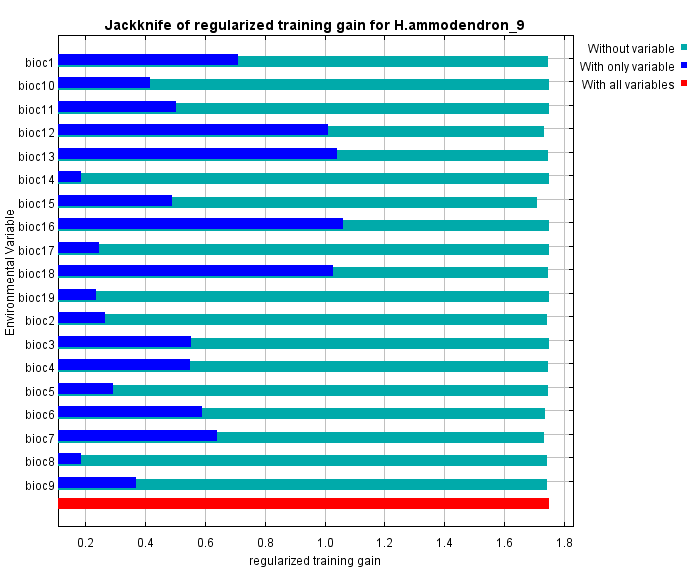

Supplement: Supplementary file 1 [file biology-13-00003-s001.zip › Figure S2 Jackknife/S254_70_jacknife.png]

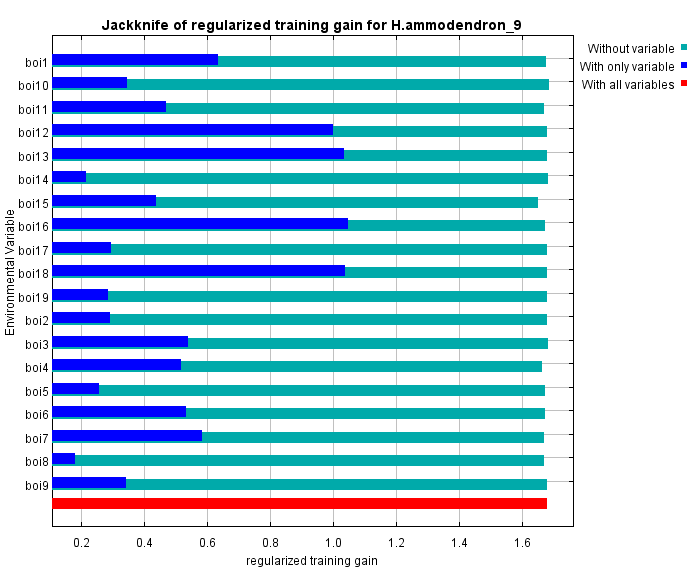

Supplement: Supplementary file 1 [file biology-13-00003-s001.zip › Figure S2 Jackknife/s585_30_jacknife.png]

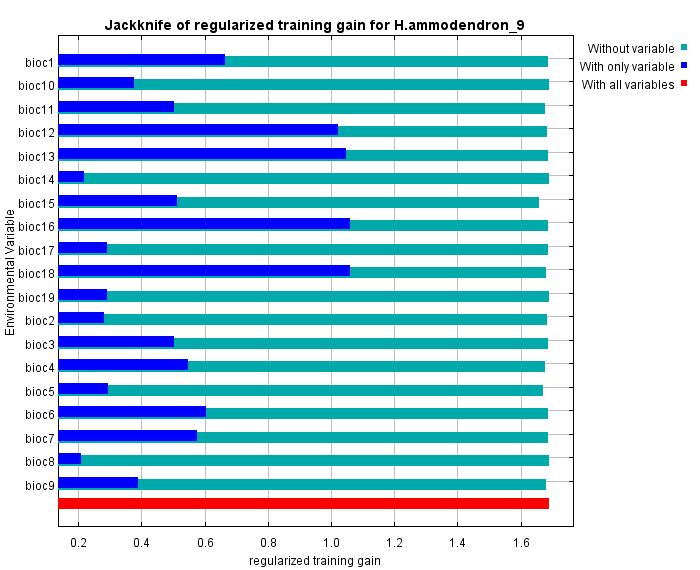

Supplement: Supplementary file 1 [file biology-13-00003-s001.zip › Figure S2 Jackknife/S585_50_jacknife.png]

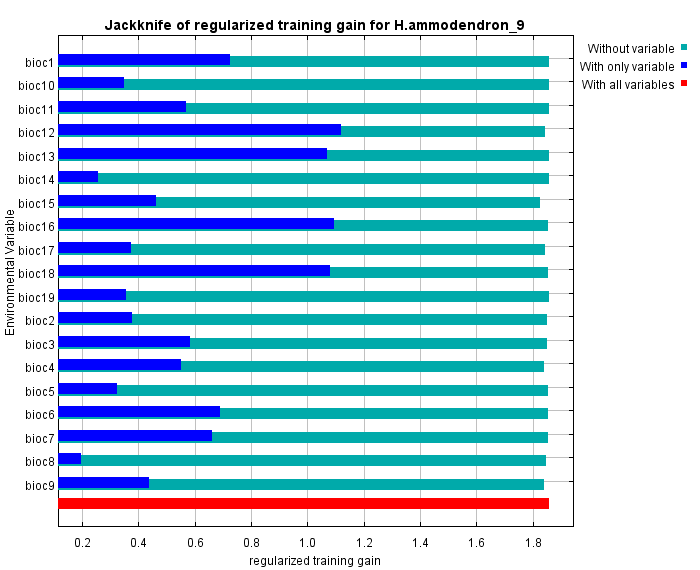

Supplement: Supplementary file 1 [file biology-13-00003-s001.zip › Figure S2 Jackknife/S585_70_jacknife.png]
